# Old and New Blood Markers in Human Colorectal Cancer

**DOI:** 10.3390/ijms232112968

**Published:** 2022-10-26

**Authors:** Jean-Luc Wautier, Marie-Paule Wautier

**Affiliations:** Faculté de Médecine, Université Denis Diderot Paris, 75013 Paris, France

**Keywords:** colorectal cancer (CRC), C-reactive protein (CRP), carcinoembryonic antigen (CEA), CEA cell adhesion molecule (CEACAM), interleukins, matrix metalloproteinases (MMPs), circulating tumor cells, circulating tumor DNA

## Abstract

Cancer is a predominant cause of mortality all over the world. Lung, prostate, and colorectal cancer are the more frequent in men while breast and colorectal have a high incidence in women. Major progress aside, some cancers are still frequent and one major issue is improvements in detection methods. Imaging techniques have a major role, but inflammatory, tumoral markers and calculated scores may contribute to the assessment of prognosis. The erythrocyte sedimentation rate (ESR), C-reactive protein (CRP), and carcinoembryonic antigen cell adhesion molecule (CEACAM) have been used for decades and do not have a clear use for diagnosis or prognosis yet. The CEACAM family includes 12 human members, and some of them have a cluster differentiation (CD). CD66 may be an interesting indicator of disease severity. Beside interleukin-6 (IL-6), the high level of which is observed in patients with a high mortality rate, other cytokines IL-17A, IL-22, and transforming growth factor -β (TGF-β) are expressed at the tumor level. The detection of circulating tumor cells has been improved but is still of undetermined value. Circulating tumor DNA (ctDNA) was recently studied in CRC stage II patients and may be helpful for chemotherapy management.

## 1. Introduction

Cancer has remained a frequent cause of death in the world, even while major progress has been made in diagnostic tools and possible treatment. Historically, surgery was the first efficacious treatment for several cancers. Unfortunately, tumor cells migrate and invade lymph nodes and other organs in the vicinity of the tumor or in different locations, such as the liver, lung, bone, or brain [1]. After the discovery of radioactivity and its effect on living cells, radioactivity was introduced as a new way to kill tumor cells. After the second world war cytotoxic drugs were administrated to patients in several ways [2]. The efficacy of chemotherapy was limited by various side effects on living cells. New strategies are being tested and are still under development, reinforcing the immune response against tumor cells or using monoclonal antibodies directed against specific antigens carried by tumor cells [3]. In recent decades, the cancer death rate decreased in men (19.2%) and in women (11.4%), since the most frequent cancers in men are lung, prostate, colorectum, and in women the two major cancers are breast and colorectum [4]. The SARS-CoV-2 pandemic may have modified the statistics and the access to information. Obviously, effort should be pursued for cancer detection and to personalize treatment. Mortality incidence was reduced in lung cancer and stabilized for breast and prostate cancer [5].

One main goal is still to detect cancer earlier and define the best protocol to cure the patients with cancer. The development of more performant techniques to detect a tumor by new systems, scanner, resonance magnetic imaging (RMI), and positron emission tomography (PET) scanning, allow an increase in the rate of detection, and to improve the follow up of patients. Since the second half of the nineteenth century several approaches have been conducted to allow for the early detection of colorectal cancer (CRC), which remained one of the most frequent life-threatening diseases. Progresses in surgery and chemotherapy have increased the life expectancy of patients with CRC mainly when the diagnosis is made at the early stage of cancer. When cells become malignant major changes in the expression of cell adhesion molecules and of cell surface glycosylation may occur. Cell adhesion is involved during the metastasis process and tumor induced immunosuppression. Several adhesion molecules such as cadherins, integrins, junctional adhesion molecules, and selectins may be modified in tumor cells [6].

The incidence of CRC was about 9% of all cancer in the population in 2012, with 1.4 million cases [7]. In 2018 the estimated incidence of CRC was of 1.8 million new cases [8]. In a study including 42 countries using cancer registry data, the incidence in ages 20–49 years was lower in India, Uganda, and Chile (3–4 per 100,000) and the highest in Korea, Australia, the USA, and Slovakia (11–13 per 100,000). In older adults the values varied between 27.5 per 100,000 in India and 192.5 per 100,000 in Slovakia. The trends of CRC incidence were stable in 14 countries and decreased in three (Austria, Italy, Lithuania). The details of incidence were described in a largely documented article [9]. CRC incidence in young adults increased in nine countries (Germany, USA, Australia, Canada, New Zealand, UK, Denmark, Slovenia, and Sweden). CRC incidence in younger adults decreased in only three countries (Italy, Austria, and Lithuania). The incidence in older adults was stable in North America, Europe, and Oceania. The main feature is the increase in CRC incidence in young adults in nine high income countries on three different continents [9].

According to TNM and modified Dukes staging, the treatment is decided, and the prognosis is correlated to the classification. Different screening systems can be used for an early diagnosis of CRC. Biological markers, used for more than half a century, are becoming one easy way for detection and evaluation of treatment efficiency. Tumor growth and cancer cell biology are modified by multiple factors. The microenvironment, including pH and oxygen supply, appears to have influence on growth invasion of tissue and metastases. These factors may also interfere with drugs used in chemotherapy [10]. A large amount of information has been obtained from animal experiments and in vitro studies [11,12]. We limited the objective of this article to human markers present in systemic blood.

The purpose of this review is to update the list of biological markers used in clinical practice or in progress, and to have a better approach for detection follow up and prognosis evaluation.

## 2. Inflammatory Markers in CRC

### 2.1. Erythrocyte Sedimentation Rate (ESR) and Hemogram

Historically, erythrocyte sedimentation rate (ESR) was routinely used as a marker of inflammation, infection, and cancer. The highest values were observed in different types of cancer and tuberculosis. Blood leukocyte count, neutrophil lymphocyte ratio, and ESR have been investigated as possible predictive indicators of prognosis in cancer patients. Complete blood cell count is routinely prescribed in patients over 50 years old. CRC may provoke gut bleeding and, consequently, reductions in the red blood cell count and hemoglobin may occur. In other cases, when an inflammatory process is induced by CRC, an increase in white blood cell count and platelet count may be observed and more specialized tests and investigation are necessary. In cases with early hepatic metastasis, modification of liver enzymes may be a first signal. Increased ESR was associated with reduced overall survival in patients with soft tissue sarcoma [13]. A poor oncologic outcome has been reported to be associated with elevated ESR in multiple myeloma and Hodgkin’s disease [14,15].

### 2.2. C-Reactive Protein (CRP)

C-reactive protein is routinely measured in patients with possible infectious or inflammatory disease. CRP is one of the major indicators of acute phase inflammatory response including cancer. CRP exists in multiple isoforms and may act as a mediator of defense response against cancer [16]. In its monomeric isoform, CRP reacts with the cell membrane and activated blood cells such as platelets and leukocytes. The pentameric isoform is the form with which blood level is measured. High levels (>10 µg/mL) were found to be associated with cancer development. CRP is of potential value for a prognosis; however, CRP values can change rapidly and a direct relationship to the stage of the extent of the disease cannot be established.

Inflammatory cytokine interleukin 6 (IL-6) and CRP blood levels are frequently correlated in pathological conditions [17]. CRP may stimulate production of interleukin 8 (IL-8). The increased level of interleukin 1 β (IL-1β) and IL-6, frequently observed in cancer, may amplify CRP production. Evaluation of CRP in cancer appears as a tool which may indicate disease severity and progression which can be useful for the therapeutic management of patients.

### 2.3. Systemic Inflammatory Score or Index

Several scores or indexes have been proposed to evaluate the prognosis of patients with colorectal cancer. In several cohorts of patients undergoing surgery, systemic inflammation reaction was evaluated by the modified Glasgow Prognostic Score (mGPS) [18,19], platelet-lymphocyte ratio (PLR), neutrophil–lymphocyte ratio (NLR) [20,21], lymphocyte–monocyte ratio (LMR) [22], and prognostic nutritional index (PNI) [23].

The modified Glasgow Prognostic Score (mGPS), an inflammation-based prognostic score, uses thresholds of C-reactive protein (>10 mg/L) and albumin (<35 g/L). The addition of neutrophil and platelet counts increased the prognostic value of the mGPS [18].

A statistical analysis using univariable analysis showed a poor overall survival in patients with preoperative NLR ≥ 5 and greater recurrence when NLR is between 1.25–5.19. According to this study NLR has a prognostic value in patients undergoing elective surgery for mismatch repair-deficient colorectal cancer [24].

According to the authors, lymphocyte–C-reactive protein ratio (LCR) is a predictor of postoperative anastomotic leakage [25]. PLR, according to a meta-analysis, may be used as an additional predictor of overall survival [26]. A meta-analysis of LMR suggests that a high ratio may be a significant predictor of better overall survival; however, it is only based on the results of retrospective studies [27]. The preoperative and postoperative low PNI were observed in the group of patients with poor survival rates [23].

Some points remained to be underlined, which limited the value of these indexes or scores. There is no quality control of the values which are issued from different laboratories, from different countries, and included a very large number of patients. The statistical analysis is sophisticated, but it is well established that we may find a correlation when the number of individuals is large enough and there are multiple parameters. If, as underlined by Rossi S. et al. [28], common factors may be responsible for the CRP, albumin synthesis, and bone marrow stimulation, the interest of multiplying the score is probably limited. Gene expression analysis or cytokine measurement may be more performant. 

### 2.4. Cytokines

Inflammatory conditions are considered to favor cancer development. Several clinical or experimental studies pointed out a relationship between interleukin-6 (IL-6) and inflammation in patients with colorectal cancer. Chronic inflammation is considered an important risk factor for cancer development in particular inflammatory bowel diseases [29]. IL-6 expression is correlated with CRC prognosis. Increased IL-6 is observed in patients with advanced CRC stage and a reduced life expectancy. According to the potential role of IL-6 through Janus kinases (JAKs) and signal transducer activator of transcription 3 (STAT3), several therapeutic approaches targeting IL-6/STAT3 have been explored [30]. The production of interferon-γ (IFN-γ) by lymphocytes has been demonstrated to limit tumor growth [31,32,33]. On the other hand, Th17 CD4, producing interleukin-17A (IL-17A) present in CRC, facilitates tumor progression in human and in experimental models [34,35]. IL-17A, IL-22, and IL-6 induce the activation of NF-κB and participate in CRC cell proliferation [36]. In 1994 Ueda T. et al. wrote that IL6 blood level was significantly elevated in different ways depending on the stage of differentiation of the adenocarcinoma [37]. A case control study and meta-analysis [38] described that IL8 may be a promising marker and found that there is heterogeneity in the European subgroup of patients with CRC. IL8 appears to have a promoting effect on cell migration through upregulation of integrin αvβ6 which is involved in CRC cell migration [39]. In addition, a meta-analysis [40] showed that higher levels of IL8 were correlated with lymphatic and liver metastasis. Interleukin10 has immunomodulatory properties. Low levels of IL10 and IL18 are found in patients with a better prognosis and a longer life expectancy [41].

Transforming growth factor-β (TGF-β) has a dual role in tumor development, it may limit cancer cell proliferation at an early stage. On tumor progress, it facilitates tumor angiogenesis and metastasis [42]. Components involved (reactive oxygen species, proteolytic enzymes, cytokines, and growth factors) in inflammatory reaction participate in the tissue reaction in the vicinity of tumors. Cancer cell dedifferentiation facilitates tumor invasion [43]. Cancer metastasis is facilitated by the enhancement of vascular permeability. In the microvasculature, circulating tumor cells tend to invade into stromal tissue. Interactions between endothelial cells (EC) and tumors are important steps mediated by different receptors or chemical structures. Monocytes may release several factors, platelet derived growth factor, vascular endothelial growth factor, and fibroblast growth factor, which may interfere with tumor growth and may enhance the expression of ICAM, VCAM-1, E Selectin, P selectin, and matrix metalloproteinases (MMPs) [44]. Tumor cell extravasation is dependent upon vascular permeability [45]. Two mechanisms may be involved in transendothelial permeability: vesicle transport and migration through endothelial cell junctions. A derived collagen tripeptide (proline–glycine–proline) promotes VE-cadherin phosphorylation and enhanced vascular permeability. Tumor cells can bind to endothelial cells and induce EC necrosis via a tumor necrosis factor (TNF) receptor family mechanism. Glycocalyx molecules, hyaluronic acid, heparan sulfate, and chondroitin sulfate limit the access of circulating tumor cells to adhesion receptors, such as intercellular cell adhesion molecule-1 (ICAM-1) or P selectin [46]. 

## 3. Tumor Markers 

### 3.1. Carcinoembryonic Antigen (CEA)

In CRC the introduction of the measurement of CEA is common but the variability limits the use as a formal diagnosis tool. The carcinoembryonic antigen (CEA), first isolated from human CRC is probably one of the major phenotypic change detectable in human cancer cells [47]. It is a glycoprotein which belongs to the immunoglobulin superfamily of proteins. The genotypic basis is incompletely known and still the goal of research [48]. Antibodies directed against CEA react with numerous proteins which were initially termed non-specific cross-reacting antigen (NCA) [49]. Glycosylated modified CEA is present in CRC. Tumor specific glycosylated CEA (Lewis X and Lewis Y) can interact with dendritic cell-specific intercellular adhesion molecule3-grabbing non integrin (DC-SIGN) [50]. 

### 3.2. CEA Structure

The human CEA gene family contains 29 genes/pseudogene. The CEA family of proteins has been proposed to be divided into three groups: CEA cell adhesion molecule (CEACAM), pregnancy-specific glycoprotein (PSG), and pseudogene group [51]. The carcinoembryonic antigen (CEA) family nomenclature has been redefined [52]. The members of the family are now named CEACAM with a number and some of them have a cluster of differentiation (CD).

### 3.3. CEACAM Family

The CEACAM joints the group of cell adhesion receptors. Attention was paid to the involvement of selectins first in inflammation and hemostasis, but also most recently in cancer metastasis [53,54]. The proteins possess an amino terminal Ig variable (IgV)-like domain. CEACAM family members (CEACAM1, CEACAM3, and CEACAM4) are connected to the intracellular domain by transmembrane helices [55]. The CEACAM are also known as CD66a to CD66e [56]. 

CEACAM1 and CEACAM3 encode transmembrane proteins while CEACAM8, CEACAM6 and CEA anchored in the cell membrane. Anoikis-mediated cell death occurred simultaneously with loss of integrins. CEACAM6 protects many cell lines from death [57,58]. CEACAM16 is a secreted form found in cochlear cells. A mutation of CEACAM 16 leads to autosomal dominant hearing loss [59].

**Figure 1:**
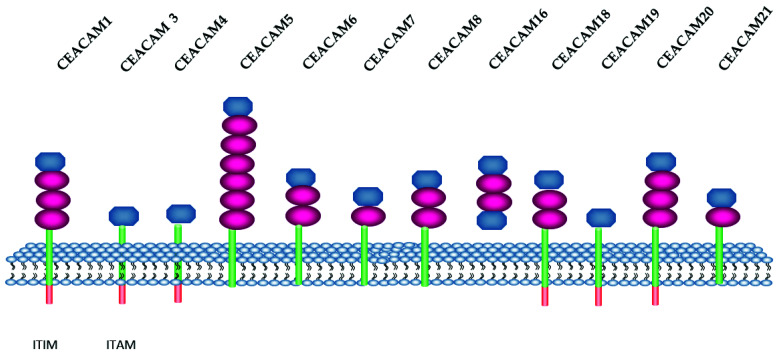
Schematic representation of human CEACAM isoforms. CEACAM belong to the immunoglobulin super family. They have at least one immunoglobulin-like domain Ig variable (IgV) and Ig constant (IgC). IgV and IgC are colored in blue and red, respectively, the cytoplasmic domain in orange and the GP-anchor in green. ITIM: immunoreceptor tyrosine-based inhibition motif. ITAM: immunoreceptor tyrosine-based activation motif (uniprot.org; proteinatlas.org) (accessed date 18 November 2021).

In humans, the CEACAM listed group consists of 12 members [60] (Figure 1), while a larger number was described in mice. In Table 1 CEACAM and CD nomenclature are listed.

### 3.4. CEACAM and Leukocytes

CEACAM1, CEACAM8, CEACAM6, and CEACAM3 (either CD66a, CD66b, CD66c, and CD66d) are expressed on human neutrophils. Anti-CD66 antibodies increased leukocyte adhesion to human endothelial cells [61]. CEACAM1 may be expressed by activated T lymphocytes. Mucin domain 3 (TIM3) is expressed on Th1 and Th17 cells [62]. TIM 3 can ligate CEACAM1 [60]. CEACAM1 interaction with TIM3 increased the inhibitory properties mediated by these receptors [63]. The interaction between CEACAM1 and TIM3 can result in T cell suppression and may play a role in regulating autoimmunity [64,65].

## 4. CEACAM and Colorectal Cancer

**Figure 2:**
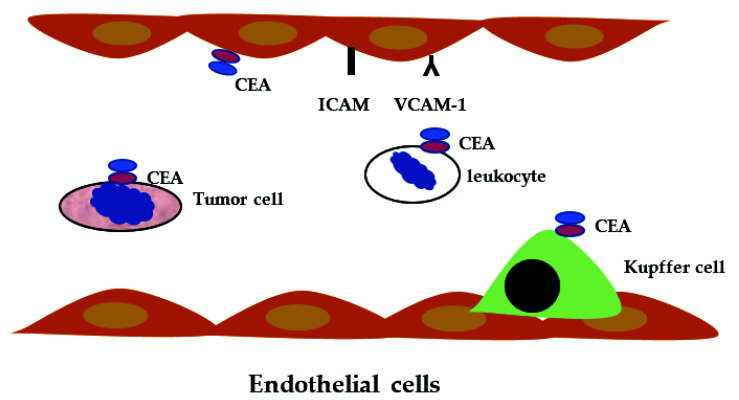
Carcinoembryonic antigen cell adhesion molecule (CEACAM) expression on cells. The CEACAM IgG superfamily may be present on normal endothelial cells, leukocytes, Kupffer cells and tumor cells (colon, breast). They are involved in cell adhesion and recognition. They participate in homophilic and heterophilic cell adhesion, facilitating migration and metastasis. CEA represents CEACAM, ICAM intercellular cell adhesion molecule, and VCAM-1 vascular cell adhesion molecule-1.

Blood CEACAM level can become increased in several pathological situations. The most developed clinical use is for cancer detection and recurrence. The use of this marker by colorectal surgeons is not unanimous. A recent review analyzed 2712 articles dealing with CEACAM in clinical practice [66]. Patients with elevated CEA had an increased frequency of colorectal cancer (CRC), 4.6% vs. 1.3%, in patients with normal CEA [67]. However, no evidence is in favor of the use of CEA for screening or diagnosis in CRC but is frequently used as an additional marker. In patients with CRC, preoperative augmented CEA is associated with a higher frequency of recurrence; a postoperative CEA decrease is a positive marker for overall survival and disease-free survival [68]. Liver metastasis is a main cause of death in patients with CRC, and CEA elevated concentration is frequently associated with liver metastasis [69]. CEA binds to the nuclear RNA binding protein M4, a Kupffer cell receptor of the liver [70]. Kupffer cells are activated after CEA binding and produced cytokines such as interleukin1 (IL-1) lL-1α IL-1β, interleukin 6 and 10, interferon-γ, TGF-β, and TNF-α. lL-1α and TNF-α stimulated expression of leukocyte adhesion molecule on endothelial cells including ICAM-1, VCAM-1, and E Selectin [71] (Figure 2).

CEACAM1 expression is reduced in the early phase of CRC [72,73]. CEACAM6 overexpression in colorectal cancer cells is correlated with the incidence of liver metastasis [74]. CEACAM6 has been shown to be important in the biology of pancreatic adenocarcinoma. CEACAM6 expression was detected in 92%, in a study including patients with pancreatic adenocarcinoma, while negative CEACAM6 expression was observed in patients with an absence of lymph node metastases and longer postoperative survival [75]. 

CGM2, now named CEACAM7, was described to be present in leukocytes and adenocarcinoma. The 2.5 kilobase transcript is strongly down regulated in colonic adenocarcinoma. This mRNA could represent a tumor specific CGM2 splice variant [76]. CEACAM 7 messenger RNA expression determined by RT PCR conducted in a single institution showed that CEACAM 7 expression is decreased in rectal cancer, and the reduction is higher in patients who developed recurrence, and may be used as a marker for patients who will benefit from adjuvant chemotherapy [77]. Using bioinformatics analysis, Bian Q et al. identified four genes associated with CRC prognosis, solute carrier family 4-member 4 (SLC4A4), glucagon (GCG), chloride (CL) channel accessory 1 (CLCA1), and CEACAM7. All four genes were downregulated in patients with CRC [78].

## 5. Biomarkers in CRC Patients

### 5.1. Carbohydrate Antigen 19-9 (CA 19-9)

CA19-9 has been tested since the last two decades of the 20th century, and the results have confirmed its usefulness as a prognostic indicator in pancreatic and gastrointestinal cancer [79]. CA 19-9 does not appear to be a useful tool for screening, due to interferences and a limited specificity and sensitivity [80].

### 5.2. Tumor-Associated Glycoprotein-72 (TAG-72)

TAG-72 evaluation in the serum has a sensitivity of 40%, which is not above that of CEA [81]. On the other hand, the surgery TAG-72 antigen-directed in CRC patients allowed a complete surgical removal of TAG-72 positive tissue. The TAG-72 positivity resection improved the immune response, increasing the life expectancy of the patients [82].

### 5.3. Tissue Polypeptide Specific Antigen (TPS)

The TPS determination, when non-associated with other markers, is difficult to evaluate as a risk factor [83].

### 5.4. Hematopoietic Growth Factors

Hematopoietic growth factors, such as granulocyte-colony stimulating factor (G-CSF) and macrophage-colony stimulating factor (M-CSF) in patients with CRC, have been found to be increased compared to normal subjects. These results have been expected and are in coherence with the blood cell count [84].

When a new blood marker can be tested, it is a good opportunity to evaluate the interest in CRC. Some markers appear to have a real value as prognostic indicators, but few of them can be used for screening, or to improve the clinical staging and the adjuvant treatment protocol adjustment. 

### 5.5. Tumor Associated Auto and Specific Antigens

Babel et al. reported 43 proteins that could distinguish between CRC patients and healthy controls. ELISA, using two of these proteins, mitogen-activated protein kinase-activated protein kinase3 (MAPKAPK3) and activin receptor2B (ACVR2B), detected CRC with a specificity of 73.9% and sensitivity of 83.3% [85]. Additional colon cancer-specific antigens (CCSA), CCSA-2, CCSA-3, and CCSA-4, were identified by proteomic analysis of structural proteins [86].

### 5.6. Matrix Metalloproteinases (MMPs)

Expression of MMPs is crucial for extracellular homeostasis. MMP1 (collagenase) genetic polymorphism is associated with CRC susceptibility. MMP13, also a collagenase, detected in the microenvironment, was increased in advanced cancer. MMP2, MMP9 (gelatinase), MMP7 (matrisylin), and MMP12 (metalloelastase), were deleterious and may have some protective effect. Tissue inhibitor of metalloproteinase-1 (TIMP-1) has been detected in plasma of CRC patients with 42–65 % sensitivity and 95% specificity [87]. TIMPs are endogenous inhibitors which limit extra cellular matrix degradation. TIMPs are involved in cell proliferation, angiogenesis, and apoptosis. Molecular inhibitors of MMPs tested in 1990 have substantial toxicity. The patient MMPs have been proposed to be useful markers for monitoring response to chemotherapy. MMPs may be of interest for the management of new therapies [88]. 

## 6. Detection of Circulating Tumor Cells in CRC Patients

To better delineate the management of patients with colorectal cancer according to the prognosis, detection of circulating tumor cells was explored using blood samples and immunotechniques. Positivity for epithelial cell adhesion molecule (EpCAM) and pan-cytokeratin (pancK) was used in a double staining [89,90]. Since the volume of blood collected is relatively limited (7.5 mL) a cell collector has been used to screen at least 750 mL of blood [91]. In a study including 80 patients with CRC, the investigators observed that the total number of circulating tumor cells was not significantly different whether they used a blood sample or the cell collector. In contrast, detection of circulating tumor cells with a cell collector was correlated with cancer stage and total survival time. The use of a cell collector appears to be a suitable technique for detection of circulating tumor cells in patients with CRC [92].

## 7. Micro-RNA and Circulating Tumor DNA (ctDNA) in CRC Patients

A new approach using micro-RNA (miRNA) as a biomarker for CRC was investigated suggesting that miRNA-106a expression was increased and miRNA-30a-3p, miRNA 145, miRNA-125a, and miRNA-133a expression was decreased in CRC tissues. miRNAs may become candidates to develop biomarkers [93].

**Figure 3:**
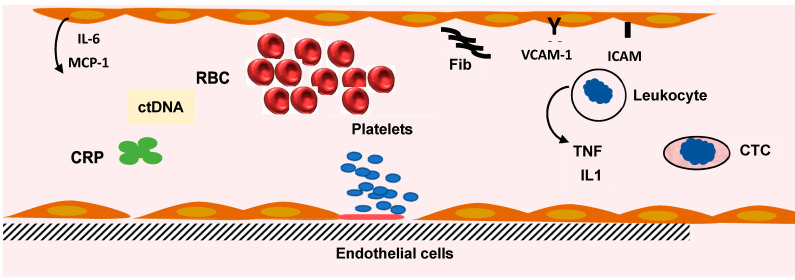
Inflammation and inflammatory markers. The defense mechanism involves leukocytes, platelets, and liver synthesis of proteins. The increase in fibrinogen (Fib) synthesis participates in the abnormal red blood cell (RBC) rheology and the increase in erythrocyte sedimentation rate. C reactive protein (CRP) blood level (pentamer) is increased. Several cytokine levels are elevated. Interleukin-6 (IL-6) is produced by endothelial cells (EC) and liver cells. Macrophage chemoattractant protein-1 (MCP1) is secreted by EC, tumor necrosis factor α (TNF), and interleukin-1β (IL1) by lymphocytes. The blood levels of cytokines are indicators of the inflammatory reaction induced by colorectal cancer. Circulating tumor cell (CTC) and circulating tumor DNA (ctDNA) can be measured in blood and used as an index of tumor progression.

DNA with tumor specific modification can be found in blood. ctDNA comes from dead tumor cells and are small fragments, including specific alteration in tumor oncogene microsatellites [94,95]. In patients with CRC treated by surgery and chemotherapy, the presence of ctDNA may represent a risk factor, but in several studies the survival time was similar with positive or negative ctDNA [96]. A meta-analysis reveals that detection panels contained genetic or epigenetic alterations. The most frequent mutations were KRAS, but other mutations can be associated. ctDNA appears to be an independent factor of prognosis [97]. Stage II CRC patients with TP53, APC, and KRAS mutations and postoperative ctDNA positivity have a reduced disease-free survival. One of the major problems is the absence of a standardized definition of ctDNA. Another difficulty is the low level of ctDNA and the difference in techniques used for the detection. In most of the studies ctDNA-positive patients have a poorer prognosis. Beside the Dukes’ staging and tumor node and metastasis (TNM) staging [98], the carcinoembryonic antigen blood level provided prognostic information (Figure 3).

Recently a multicenter Australian investigation explored whether or not ctDNA positivity in stage II CRC patients’ blood can be used as an indicator for the response to adjuvant therapy. Patients after surgery were divided into two groups according to ctDNA guided management or standard management. A group of 302 patients were in the ctDNA guided management and 153 in the standard management. Patients received chemotherapy by oxaliplatin or fluoropyrimidine chemotherapy. In the group of ctDNA positive patients treated with adjuvant therapy, the recurrence free survival (RFS) was 86.4% at 3 years. On the other hand, ctDNA negative patients without treatment at 3 years, the RFS was observed in 92.5%. This study indicates that ctDNA may be an additional parameter for adjuvant therapy decision [99]. 

The results of PATHFINDER, a multicenter study, multicancer early detection (MCED) were reported. The study was conducted in 6621 patients and the ctDNA analyzed in blood samples. The MCED test detected cancer signal in 1.4%. The specificity was estimated at 99.1%. This preliminary report may be of great potential interest, when completely published.

## 8. Conclusions

Besides the clinical staging of CRC, additional information may be of particular interest to evaluate the benefit of chemotherapy versus the side effects. One approach has been achieved using genetic markers. Circulating tumor cells and circulating tumor DNA may be new tools to evaluate the prognosis and the need of treatment. New targeted therapies using RNA silencers, monoclonal antibodies, have to be tested on larger trials, and the use of criteria accessible by blood sampling may find a space in the management of patients with CRC.

## Figures and Tables

**Table 1:** CEACAM Family Members (uniprot.org; proteinatlas.org) (accessed date 18 November 2021).

Name	Amino Acid	Molecular Weight (kDa)
CEACAM1, CD66a	526	57.6
CEACAM3, CD66d	252	27.1
CEACAM4, CGM7	244	28.9
CEACAM5, CD66e	702	76.8
CEACAM6, CD66c	344	37.2
CEACAM7, CGM2	265	29.3
CEACAM8, CD66b	349	38.1
CEACAM16	425	45.9
CEACAM18	384	43.3
CEACAM19	300	32.6
CEACAM20	596	65.8
CEACAM21	293	32.3

## Data Availability

Not applicable.
